# Toll-Like Receptors in Secondary Obstructive Cholangiopathy

**DOI:** 10.1155/2011/265093

**Published:** 2011-10-27

**Authors:** A. G. Miranda-Díaz, H. Alonso-Martínez, J. Hernández-Ojeda, O. Arias-Carvajal, A. D. Rodríguez-Carrizalez, L. M. Román-Pintos

**Affiliations:** Centro Universitario de Ciencias de la Salud, Universidad de Guadalajara, Guadalajara, 44340 JAL, Mexico

## Abstract

Secondary obstructive cholangiopathy is characterized by intra- or extrahepatic bile tract obstruction. Liver inflammation and structural alterations develop due to progressive bile stagnation. Most frequent etiologies are biliary atresia in children, and hepatolithiasis, postcholecystectomy bile duct injury, and biliary primary cirrhosis in adults, which causes chronic biliary cholangitis. Bile ectasia predisposes to multiple pathogens: viral infections in biliary atresia; Gram-positive and/or Gram-negative bacteria cholangitis found in hepatolithiasis and postcholecystectomy bile duct injury. Transmembrane toll-like receptors (TLRs) are activated by virus, bacteria, fungi, and parasite stimuli. Even though TLR-2 and TLR-4 are the most studied receptors related to liver infectious diseases, other TLRs play an important role in response to microorganism damage. Acquired immune response is not vertically transmitted and reflects the infectious diseases history of individuals; in contrast, innate immunity is based on antigen recognition by specific receptors designated as pattern recognition receptors and is transmitted vertically through the germ cells. Understanding the mechanisms for bile duct inflammation is essential for the future development of therapeutic alternatives in order to avoid immune-mediated destruction on secondary obstructive cholangiopathy. The role of TLRs in biliary atresia, hepatolithiasis, bile duct injury, and primary biliary cirrhosis is described in this paper.

## 1. Innate Immunity

Innate immunity acts, responds, modulates, and provides defense against viral and bacterial infections through trans-membrane cell receptors encoded in the germ line, among them are the Toll-like receptors (TLRs). As part of the innate immune response, there are several endogenous antibiotics against *bacterial *(lactoferrins, lysozymes, and defensins), and *viral *infections (interferon-1*β* and MxA-protein); cytokines and chemokines also participate significantly in acquired immunity. Biliary epithelial cells secrete immunoglobulin A in relation to pathogen-associated molecular patterns (PAMPs). When biliary epithelium gets in touch with a pathogen, it is then activated by PAMPs and induces different mechanisms to prevent or limit tissue damage by means of Toll-like receptor-3 (TLR-3), which in turn stimulates transcriptional nuclear factor-*κ*B (NF-*κ*B) after production of antiviral interferon-1*β* (IFN-1*β*) [1]. The bowel has to react to pathogens while keeping tolerance to normal commensal flora. TLRs play an important role in this delicate balance because they induce activation of innate and adaptive immune response, that is why a low expression of TLRs is found on epithelial gut cells to maintain tolerance to microorganisms [2].

## 2. Hepatobiliary Tract Innate Immunity

Many symptomatic infectious diseases of the biliary tract are characterized by neutrophil migration across the epithelium and accumulation within the lumen. Transepithelial migration of neutrophils represents an important factor in mucous epithelium defense of inflammatory diseases. When inflammatory reaction starts on biliary epithelium, neutrophils traverse vascular endothelium to lamina propria and reach epithelium. Transepithelial migration of neutrophils can be initiated by stimulation of interleukin-8 (IL-8) as a consequence to host-pathogen interaction and regulated by early events in response to transepithelial migration of CD11b/CD18-*β*2 integrin. Pathogen microorganisms by themselves can release bacterial peptides such as N-formate, potent neutrophil chemoattractant in transepithelial migration [3]. 

Biliary innate immunity is actively involved in inflammation by secreting cytokines (IL-1*β*, tumor necrosis factor-alpha (TNF-*α*), and IL-6), chemotactic cytokines (monocyte chemotactic protein (MCP-1), inhibitory factor macrophage migration (MIF)), proinflammatory proteins, reactive oxygen species, and adhesion molecules and expression of receptors that recognize microorganisms, such as TLRs in biliary epithelium cells, which provides protection against bacterial and viral infections. On the other hand, biliary epithelium is constantly exposed to PAMPs that do not trigger inflammatory responses, denominated as immune tolerance, which maintains homeostasis between the body and commensal bacterial flora, thus avoiding excessive damage to the epithelium [4]. It has been shown certain cross-tolerance of biliary epithelial cells, monocytes, and intestinal epithelial cells [5] to lipopolysaccharides (LPSs)—TLR ligands-4—after their exposure for 24 h, observed by overexpression of IRAK-M which interferes in the association between IL-1 receptor-associated kinase (IRAK-1) and myeloid differentiation factor 88 (MyD88) [6].

## 3. Toll-Like Receptors

Thirteen homologous recipients to TLRs have been described in mammals of which 10 have been described in humans [7]. TLRs are part of pattern recognition receptors (PRRs), well-conserved multigene family in humans which plays an important role in innate and adaptive immunity by recognizing unique structural components of bacteria, fungi, viruses, and damage-associated molecular patterns (DAMPs). TLRs are transmembrane receptors expressed in most immune cells including polymorphonuclear and epithelial cells. They have an extracellular 24-to-29-aminoacid domain with leucine-rich regions (LRRs), one or two cysteine-rich regions, and a 200-aminoacid intracellular domain, similar to that described in the family of interleukin-1 receptors (IL-1R), called Toll/IL-1R (TIR), which performs signal transduction [8]. TLRs can also act as intracellular receptors related to individual characteristics of pathogen components, such as nucleic acids, proteins, lipids, and carbohydrates. Binding substrates couple to TLRs leading to signaling and activation of innate and adaptive inflammatory response, which represents a bridge between both systems since the activation of innate immune system induces phagocytosis, opsonization, and production of inflammatory mediators, blocking the spread of the pathogen. After recognizing their ligands, TLRs expressed on antigen-presenting cells (APCs) are activated and induce molecules involved in presenting antigenic peptides on the surface. In the major histocompatibility complex (MHC), peptides are recognized by antigen-specific T cells, thus joining the innate and adaptive immune response [9]. PAMP can be compromised by TLRs, as they are basic characteristics of the microorganisms that cannot be modified by genetic mutations in a short time [10]. Description of human TLRs is provided next, and Table 1 contains their main characteristics.

### 3.1. Toll-Like Receptor-1, -2, and -6

TLR-1 is a member of the innate immune system family receptors [11]. It recognizes PAMP specifically from Gram-positive bacteria, also known as CD281. TLR-1 inhibits phenol-soluble modulin response mediated by TLR-2 secreted by *Staphylococcus epidermidis* and participates in the recognition of soluble factors released by *Neisseria meningitidis*. 

Specific activation by homo- or heterodimerization of TLR-2 on hepatitis C core proteins along with TLR-1 and TLR-6 and reduction of cytokines production by blocking these receptors have been reported, suggesting that hepatitis C proteins activate innate immune reckoning [12]. 

TLR-6 was identified on knockout mice as a coreceptor necessary for recognition of diacylated lipopeptides, while TLR-1 forms heterodimers with TLR-2 and recognizes triacylated lipopeptides structures. TLR-1 gene is expressed in all cells at high levels and recognizes peptidoglycan in accordance with TLR-2 as heterodimer, which is found on the surface of macrophages and neutrophils [13]. 

TLR-1, TLR-6, and TLR-10 can form heterodimers with TLR-2 by increasing the specificity of their ligands [14, 15]. It has been shown that TLR-2 plays an important role in lipid transport through lipoproteins uptake. It appears that TLR-2 deficiency protects from nonalcoholic fatty liver disease and probably modifies the signaling pathway of TLR-4 [16]. TLR-2 activation requires the presence of leucine-rich cofactors such as CD14. These receptors are located on cell membrane and they recognize peptidoglycan, lipoteichoic acid and lipoproteins of Gram-positive bacteria, spirochetal LPS, zymosan from fungi, *Treponema maltophilum* glycopeptides, glucoinositol phospholipid from *Trypanosoma cruzi* and modulin [14]. TLR-6 recognizes *Mycoplasma* and *Borrelia burgdorferi* lipoproteins along with TLR-2 and also recognizes differences between acylated lipopeptides of pathogen microorganisms [17, 18].

### 3.2. Toll-Like Receptor-3

TLR-3 reacts to dsRNA from viruses and apoptotic and/or necrotic cells. Dying cells can activate TLR-3 by an activating ligand. TLR-3 in the liver may mediate innate activity and inflammation and induces type I IFN production during viral infections. Finally, TLR-3 is overexpressed in the endosome of dendritic cells [19].

### 3.3. Toll-Like Receptor-4

TLR-4 was the first one to be discovered and is considered the most important because it is the first to respond to LPS ligand, also best known for his sensitivity to detect LPS in Gram-negative bacteria membrane. TLR-4 constitutes the largest component of the LPS recognition receptor complex (coreceptors CD14 and MD-2) [20, 21], and its signaling can be activated by some cellular components of endogenous ligands which are released or increased during tissue injury and matrix degradation (DAMPs) [22]. This receptor is involved in LPS endotoxin response that occurs on the cell surface of Gram-negative bacteria by activating signaling pathways, resulting in synthesis of inflammatory cytokines and type I IFN. Its activation requires the presence of cosignaling receptors MD-2, although some TLR-4 can generate the signal in absence of CD14 [23].

### 3.4. Toll-Like Receptor-5

TLR-5 recognizes the bacterial component flagellin protein of *Salmonella typhimurium*. Flagellin is a potent activator of systemic inflammation in humans, and its serum levels are positively correlated with clinical severity in presence of bacteremic shock by playing an important role in the immunopathogenesis of inflammatory bowel disease. Flagellin is also capable of activating apoptotic signaling pathways and proinflammation and can lead to activation of innate immunity [24].

### 3.5. Toll-Like Receptor-7

TLR-7 is involved in recognition of imidazoquinolines. These receptors induce type I IFN production during viral infections and are expressed on endosome of dendritic cells, human plasmacytoid cells, myeloid cells, and monocyte-derived cells; thereby, its expression on different body systems depends on the number of dendritic cells held by each organ.

### 3.6. Toll-Like Receptor-8

TLR-8 is activated in viral infections producing type I IFN. It belongs to a family of endosomic recognition pattern receptors capable of producing type I IFN MyD88-dependent and detect pathogen-derived nucleic acids. Human monocytes constitutively express TLR-8 [25].

### 3.7. Toll-Like Receptor-9

TLR-9 recognizes bacterial DNA with unmethylated motifs. Apoptotic bodies containing microkernels are a source of DNA. The unmethylated motifs of cytosine-phosphate-guanosine- (CpG-) DNA are recognized by TLR-9. TLR-9-deficient mice manifest less liver injury in response to poisoning by carbon tetrachloride, bile duct ligation, acetaminophen and fatty liver hepatitis model. TLR-9 activates different signaling cascades resulting in generation of myofibroblasts by hepatic stellate cells (HSCs), collagen type 1, and overexpression of the profibrogenic cytokine TGF-*β* because its effects can be blocked by TLR-9 antagonists, suggesting activation of the innate immune response within the HSC. The TLR-9 responds to viral infections by producing type I IFN [26].

### 3.8. Toll-Like Receptor-10

TLR-10 is an orphan members of human TLRs. It shares a common *locus* on chromosome 4p14 [27]. In experimental models TLR-10 is predominantly expressed in immune cells-rich tissues such as small bowel, stomach, thymus, peripheral blood lymphocytes, lymph nodes, and tonsils [28]. In co-immunoprecipitation studies an association with TLR-1 and TLR-2 through extracellular domains has been demonstrated, and also an important role by inducing production of IFN type I on plasmacytoid dendritic cells. TLR-10 in humans recognizes conserved motifs of bacteria and viruses, resulting in inflammation mediated by TLRs activating transcription factor NF-*κ*B and IFN regulatory factor [29].

## 4. TLRs in Liver Damage and Inflammation

Different cholangiopathies result from biliary tract dysfunction, and they can be divided into autoimmune, genetic, infectious, drug-induced, or ischemic, and on the other hand by complete biliary obstruction with intra- and extrahepatic bile stasis; therefore the pathogenesis of some cholangiopathies remains unclear, which results in limited treatments. 

TLRs are expressed on the liver, an organ that receives blood from the portal circulation of lower extremities and bowel with frequent exposure to PAMPs of invading microorganisms and antigenic components of the diet; therefore, healthy liver can develop immunological tolerance [30]. PAMPs are detected in normal and pathological bile indicating that biliary epithelial cells are exposed to bacterial components under physiological and pathological conditions [31]. Hepatocytes express mRNA for all TLRs [32, 33] but only TLR-2 and TLR-4 show functional activity of endotoxemia clearance in circulation [34]. Both parenchymal and nonparenchymal liver cells express TLR-4 and are actively involved in the response to different types of liver injury. Liver injury, regardless of etiology, including viral hepatitis, alcoholic and nonalcoholic disease, autoimmune liver diseases induced by drugs, and other diseases that prevent free passage of bile into duodenum with complete intra- or extrahepatic bile duct obstruction, is associated with increased hepatic exposure to bacterial products. When liver sinusoidal endothelial cells are exposed to low levels of LPS, it can result in loss of expression and desensitizing of TLR-4 [35]. 

## 5. Hepatic Immune Response

Liver immune response is complex due to competition of many cell populations, including lymphocytes binding with natural killer (NK) and CD8+ T cells which may be unusually abundant. Dendritic cells (DCs) and activated hepatic stellate cells (HSCs) show TLR-4 signals which participate in hepatic inflammation and fibrogenesis. These cells can respond to TLRs signals and act as antigen-presenting cells for T cells. TLRs have been found in the liver when infected with viral hepatitis B or C, and also in animal models of bile duct injury induced by ligation, partial hepatectomy, drug toxicity (acetaminophen), and ischemia-reperfusion. TLR-4 is specific for LPS by transferring the signal inside the cell and promoting translocation of NF-*κ*B into the nucleus with transcriptional activation of chemokines-encoding genes and pro- and anti-inflammatory cytokines [36].

## 6. TLRs in Liver Fibrosis

**Figure 1:**
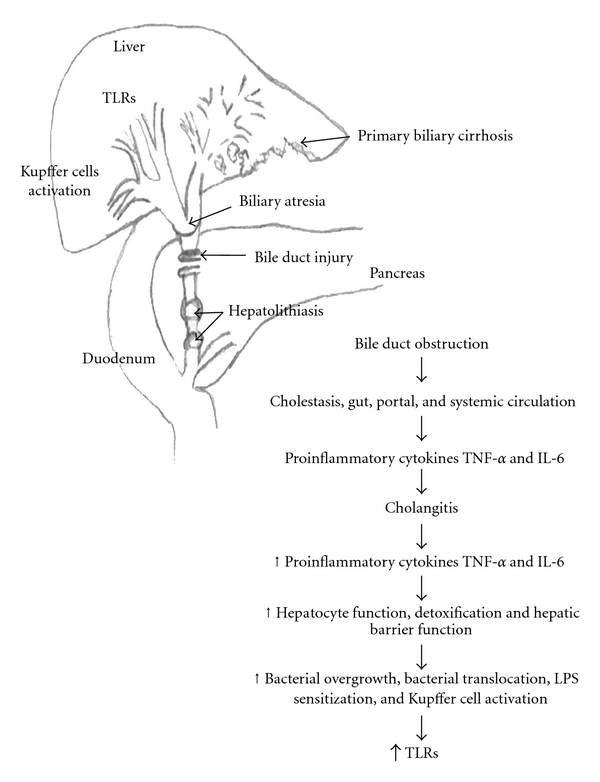
Toll-like receptors on secondary obstructive cholangiopathies.

Any immune or inflammatory liver disease activates Kupffer cells; these can trigger production of inflammatory cytokines (IL-6, IL-8, and TNF-*α*) with subsequent activation of HSCs, promoting fibrogenesis (excessive deposit extracellular matrix proteins) and TLRs participation, which results in cirrhosis and liver failure. The activation of HSCs is controlled by many cytokines and growth factors, including TGF-*β* which is considered the main inductor on transformation of HSCs, while growth factor platelet-derived (PDGF) plays a critical role in stimulating the proliferation of HSCs. Recent studies suggest that TLR-4 participates in the production of hepatic fibrosis by activation of HSCs by TGF-*β* [37]. Yokoyama et al. showed that TLR-2 and TLR-4 are capable of mediating innate immune system on intrahepatic biliary epithelial cells. Furthermore, increased expression of TLR-4 on biliary epithelium in primary biliary cirrhosis [38] after bile duct ligation on experimental studies [37] suggests TLRs role in the development of chronic inflammation and liver fibrosis (Figure 1). 

It has been reported evidence of an association of hepatic fibrosis and angiogenesis by TLR-4 through MyD88 on liver endothelial cells [39]. TLR-4 has been identified as one of the seven genes associated with increased risk of developing cirrhosis in patients infected with Hepatitis C virus (HCV) [40, 41]. TLR-3 bounded to poly I : C induces activation of NK cells [42] which can destroy HSCs, and produces IFN-gamma to induce apoptosis and inhibit proliferation of HSCs leading to inhibition of liver fibrosis [43, 44].

## 7. TLRs in Liver Regeneration

Liver regeneration is a complex process orchestrated by several signaling pathways induced by cytokines, growth factors, and hormones. Experimental studies suggest that loss of liver tissue or cell damage is caused by innate immune response to liver regeneration. The role of TLR-4 signaling in liver regeneration has been widely investigated through MyD88 [34] on alcohol injury and development of hepatic steatosis [45, 46]. TLR-3 on bladder epithelial cells not only plays an important role by protecting against viral infection through secretion of IFN-gamma [47] but also causes apoptosis of infected liver cells and has been associated with pathogenesis of biliary atresia [48]. It also inhibits liver regeneration of damaged hepatocyte apoptosis and interrupts the cell cycle (cyclin dependent (e.g., cyclin D1)) through activation of transducers and activators of transcription signal-1 (STAT-1) [49].

## 8. TLRs in Liver Diseases

### 8.1. Secondary Obstructive Cholangiopathy

Human bile is sterile under normal conditions. Acute cholangitis is a biliary tract infection characterized by fever, right upper quadrant abdominal pain, and jaundice [50]. Its etiology is diverse, including choledocholithiasis, biliary atresia, and complete obstruction of the bile ducts after cholecystectomy. For the cholangitis to develop bile stasis is required with increased bladder pressure and presence of microorganisms in bile ducts, being the most common pathogens Gram-negative bacteria [51]. If not resolved soon, it can evolve to sepsis and systemic inflammatory response syndrome leading to high morbidity and mortality with inadequate hepatic and systemic response in presence of LPS [52]. 

Biliary epithelial cells are immunologically potent. In presence of bile duct inflammation these cells secrete cytokines which express immunity receptors further recognizing microbes through PRRs. TLRs are the better recognizer of PAMPs. Upon exposure to LPS, immunity cells are activated and they transduce intracellular adaptative signals associated with TLRs, induce NF-*κ*B expression, and secrete proinflammatory cytokines.

It has been confirmed by immunohistochemistry that TLR-1/TLR-5 are distributed diffusely in intrahepatic biliary tract on healthy human liver. Biliary epithelial cells possess TLRs signaling systems which actively participate in cellular immunity [20, 53]. Increased expression of TLR-4 in biliary epithelium after experimental bile duct obstruction promotes chronic inflammation and hepatic periportal fibrosis which depends on the increase of LPS binding proteins [10]. TLR-4 role in acute cholangitis has not yet been fully elucidated. 

Upon epithelium exposure to pathogen particles, adaptive immunity induces resistance against microorganisms by rearrangements of genes encoding T cell receptors producing high levels of different receptors in response to attacks by pathogens. It is well known that acquired resistance is not transmitted vertically and reflects the individual experience of each organism to previous infections. In contrast, innate immune receptors recognize antigens by specific PRR receptors which are vertically transmitted by germ cells where antigen recognition induces an immediate inflammatory response to control infection by opsonization, chemotaxis, and activation of leukocytes. As mentioned previously, TLRs induce inflammatory response against microorganisms through activation of NF-*κ*B as it induces transcription of many genes including cytokines such as TNF, IFN, IL-2, IL-8, and IL-12 and defensins within minutes after pathogens injury causing inflammation in order to destroy the infection and generate immunological memory against [54]. 

Knowing the intricate inflammation mechanisms of epithelium bile duct injury, it is essential to develop alternative therapies aimed to prevent immune-mediated destruction of biliary tract associated with cholangiopathy. Four cholangiopathies characterized by secondary biliary obstruction will be discussed briefly, capable of producing secondary biliary cirrhosis. Liver cirrhosis will also be addressed, and finally some alternative management related to TLRs.

### 8.2. Biliary Atresia

Biliary atresia is characterized by a progressive inflammatory sclerosing obstructive cholangiopathy which appears within the first afterbirth months. The precise etiology and pathogenesis remains unknown; it could be caused by viral infection with *Reoviridae* (*reovirus* type 3, type C *rotavirus*, *adenovirus*, *Cytomegalovirus*, *Estein-Barr,* and others), even though biopsy results obtained during Kasai procedure are contradictory [55]. Immunohistochemical analysis of early innate immune response proteins with molecular markers for Mx is highly sensitive to type I interferon, and they are also reported as positive in hepatocytes and intrahepatic bile ducts in biliary atresia, suggesting the presence of viral infections [56, 57]. It is possible that biliary endothelial cells are targets of these viruses producing atresia. Even when survival of patients improves significantly with Kasai procedure, more than half of affected children will develop cirrhosis and will require liver transplantation [58]. 

A previous study published by Huang et al. found significantly increased expression of TLR-5 and MxA mRNA in liver biopsy from early biliary atresia. These results imply the participation of TLR-7 and type 1 interferon signaling in the pathogenesis of biliary atresia, specifically on early stages and its association with overproduction of IL-8 [59]. Despite the above mentioned, much remains to be elucidated regarding TLRs and biliary atresia.

### 8.3. Hepatolithiasis

It is considered a bile duct disease histologically characterized by formation of gallstones and chronic proliferative cholangitis. Reported incidence in western countries is about 1%, although it is not rare in Asia (including Japan). Its natural history can lead to development of biliary cirrhosis, portal hypertension, and liver failure, with risk of cholangiocarcinoma in approximately 10% of patients. Bacterial infections of biliary tract and presence of cholestasis have been implicated as the main etiopathogenic factor of lithogenesis in patients with calcium bilirubinate stones, and *Escherichia coli* (*β*-glucuronidase) is the most frequently isolated bacteria in bile ducts. On the other hand, DNA of *Campylobacter* species has been reported by PCR in bile and bladder biopsies from patients with hepatolithiasis [60]. It is speculated that these bacteria in biliary epithelium influence the emergence and development of cholangitis and lithogenesis, through interaction of PAMPs and TLRs, although its mechanism remains unclear.

### 8.4. Bile Duct Injury after Cholecystectomy

TLRs role in patients with bile duct injury after cholecystectomy has not been elucidated. Experimental studies on animals submitted to bile duct ligation have been reported; however, human reactions are not analogous to those on experimental models. Secondary obstructive congenital or acquired cholangiopathies generate significant morbidity and mortality in both children and adults because cholangiocytes proliferate at the final stage of the disease, resulting in bile duct destruction, liver fibrosis, following cirrhosis and finally liver failure. We have to look into the behavior of TLRs in serum and liver tissue biopsies from patients with biliary atresia, hepatolithiasis, biliary injury previously undergone cholecystectomy, and primary biliary cirrhosis in order to describe the role of TLRs for planning alternative surgical or biomolecular treatment strategies.

### 8.5. Cirrhosis

Patients with liver cirrhosis have chronic endotoxemia, elevated serum TNF-*α*, IL-1*β*, and IL-6 due to activation of Kupffer cells, and high baseline levels of LPS-induced TLR-4 expression. However, regulation of TLR-4 is altered in monocytes from cirrhotic patients which produce frequent bacterial infections [61].

### 8.6. Primary Biliary Cirrhosis

Primary biliary cirrhosis has an immune etiology and produces biochemical and structural alterations of the liver acting as an obstructive cholangiopathy. Patients with chronic liver diseases regardless of etiology are likely to develop liver fibrosis that can lead to cirrhosis with consequent complications such as portal hypertension and/or liver failure. All chronic liver diseases including cholangiopathy generate a constant process of fibrosis where the innate immune system and activation of monocytes and NK cells induce chronic inflammation [62]. Primary biliary cirrhosis is characterized by destruction of intrahepatic bile ducts through autoimmune mechanisms apparently caused in response to inflammatory cytokines. There are also several reports suggesting that pathogenic microorganisms are involved in the pathogenesis of the disease [63]. TLRs and type I IFN play an important role in innate and adaptive immune response, and as mentioned before type I IFN has the ability to induce maturation of DC during antigen presentation and direct cellular differentiation by MHC and induces antibody production. Type 1 IFN may be implicated in the pathogenesis of autoimmunity in primary biliary cirrhosis and is induced by TLR-3, TLR-4, TLR-7, and TLR-9 [64]. An incidence increase of recurrent urinary tract infections in patients with primary biliary cirrosis has been reported; furthermore, other studies support the association between urinary or vaginal infections with endotoxemia in bile ducts due to proteins, nucleic acids, or PAMPs that induce inflammation. Some authors speculate that these may be responsible for cholangitis that occurs in primary biliary cirrhosis [65].

### 8.7. Management Alternatives for Secondary Biliary Cholangiopathy

#### 8.7.1. TLRs Blockade

Specific activation of TLR-2 in hepatitis C and homo- or heterodimerization with TLR-1 or TLR-6 has been shown; also, when these are blocked a significant decrease on production of proinflammatory cytokines appears, suggesting that some proteins produced by hepatitis C activate innate immune [12]. TLR-9-deficient mice manifest less liver injury in response to carbon tetrachloride, bile duct ligation, acetaminophen, and steatohepatitis. Overexpression of TGF-*β* can be blocked by TLR-9 antagonists [26]. TLR-2 deficiency appears to play a protective role in induction of nonalcoholic fatty liver disease and probably modify TLR-4 signaling [16]. TLR-2 and TLR-4 induce endotoxemia clearance in plasma [33]. TLR-3 inhibits liver regeneration of damaged hepatocyte apoptosis and seize cell cycle through signaling transcription activators-1 (STAT-1) [49]. Therefore, it is important to perform clinical trials and experimental studies on protein-deficient animals in order to understand the biochemical and immunological mechanisms that may be susceptible to modification to improve pathogenic response to microorganisms in secondary obstructive cholangiopathy, thus, preventing liver fibrosis, cirrhosis, and portal hypertension.

#### 8.7.2. TLRs Stimulation

TLR-1, TLR-2, TLR-3, TLR-7, TLR-8, TLR-9, and TLR-10 induce type 1 IFN production during viral infections [19]. TLR-3 poly I : C-linked induces activation of NK42 cells which destroy HSCs and IFN-gamma production by inducing apoptosis and inhibition of HSCs proliferation, leading to liver fibrosis restrain [43, 44]. TLR-3 in biliary epithelial cells protects against viral infections by secretion of IFN-gamma, inducing apoptosis of infected hepatocytes, as reported in biliary atresia. Moreover, there are some antimicrobial peptides produced by epithelial cells called human *β*-defensins induced by inflammatory stimuli and produced specifically on infection site. *β*-defensins interact on bacteria membrane through exchange of hydrophobic amino acids. They are expressed mainly on epithelial cells and have a broad activity spectrum against Gram-positive and Gram-negative bacteria, fungi, and certain viruses [66]. All of the above are strong research possibilities; however, it would be worthwhile to develop prospective clinical trials to be carried out in bile cultures for searching Gram-positive and Gram-negative bacteria, anaerobic bacteria, viruses, and fungi, as well as evaluating TLRs and *β*-defensins behaviour in secondary obstructive cholangiopathy in patients at hospital admission and at hospital discharge considering as variable the administration of antibiotic therapy. Agonists of TLR-7 and TLR9 are under consideration in the management of hepatitis C [67]. The better understanding of the role played by TLRs in secondary obstructive cholangiopathy would offer more and improved therapeutic options aimed to prevent adverse outcomes for these pathologies.

## Figures and Tables

**Table 1:** Toll-like receptor(TRLs) characteristics. Some TLRs recognize proteins, lipids, and nucleic acids from RNA or DNA of pathogens by inducing innate immune response. Stimulation of TLRs starts with the activation of intracellular signals resulting in secretion of proinflammatory cytokines such as type 1 IFN, TNF-*α*, and IL-6 depending on the causal agent.

	Location	Function	Stimulates	Inhibits or interferes
Toll-like receptor (TLR)	(i) Transmembrane receptors expressed in most immune cells including polymorphonuclear and epithelial cells (ii) Intracellular receptor function	(i) Induction of phagocytosis, opsonization, and production of inflammatory mediators,	(i) Innate and adaptive inflammatory response	(i) The spread of the pathogen

TLR-1	(i) Expressed on the surface of all cells at high levels (ii) Recognizes peptidoglycan (iii) Found on macrophages and neutrophils	(i) Recognition of PAMP from Gram-positive bacteria (ii) Reduction of cytokines production	(i) Heterodimers increasing the specificity of their ligands	(i) Phenol-soluble modulin

TLR-2	(i) Expressed on cell membranes surface (ii) Found on macrophages and neutrophils	(i) Lipid transport through lipoproteins uptake (ii) Formation of heterodimers (iii) Recognition of triacylated lipopeptides, structures of peptidoglycan, lipoteichoic acid and lipoproteins of Gram-positive bacteria, spirochetal LPS, zymosan from fungi, *Treponema maltophilum* glycopeptides, glucoinositol phospholipid from *Trypanosoma cruzi* and modulin	(i) Requires the presence of leucine-rich cofactors such as CD14	(i) Deficiency protects from nonalcoholic fatty liver disease

TLR-3	(i) Intracellular expression on endosomes of myeloid DCs	(i) Reaction to dsRNA from viruses and apoptotic and/or necrotic cells	(i) Type I IFN production during viral infections and NF-*κ*B	(i) Antagonist

TLR-4	(i) Expressed on all cell surface	(i) First to respond to LPS ligand (ii) Detection of LPS in Gram-negative bacteria membrane	(i) IRAK-M (ii) Cellular components (iii) Endogenous ligands which are released or increased during tissue injury and matrix degradation (iv) Synthesis of inflammatory cytokines and type I IFN	(i) IRAK-1, MyD88, and NF-*κ*B

TLR-5	(i) Expressed on all cell surface	(i) Recognition of bacterial flagellin protein of *Salmonella typhimurium * (ii) Induction of inflammatory responses via a MyD88-dependent or independent pathway	(i) Initiation or amplification of Th2-type (ii) Innate immunity (iii) Apoptotic signaling pathways and proinflammatory	(i) I-*κ*B degradation (ii) NF-*κ*B activation for soared tissue

TLR-6	(i) Expressed in myeloid and monocyte-derived dendritic cells (DCs) (ii) Not expressed on plasmacytoid DCs (pDCs)	(i) Recognition of *Mycoplasma* and *Borrelia Burgdorferi * (ii) Differences between acylated lipopeptides of pathogen microorganisms (iii) Formation of heterodimers with TLR-1, TLR-2, and TLR-10	(i) Specificity of ligands from TLR-1, TLR-2, and TLR-10	(i) Antagonist

TLR-7	(i) Expressed on endosome of dendritic cells, human plasmacytoid cells, myeloid cells, and monocyte-derived cells	(i) Recognition of imidazoquinolines	(i) Type I IFN production during viral infections (ii) Agonists of TLR-7	(i) Antagonists

TLR-8	(i) Intracellular expression	(i) Detection of pathogen-derived nucleic acids (ssRNA)	(i) Type I IFN MyD88-dependent	(i) Antagonists

TLR-9	(i) Intracellular expression	(i) Recognition of bacterial DNA with unmethylated motifs (ii) Activation in viral infections (iii) Recognition of DNA from viral infections	(i) Type I IFN	(i) Antagonists

TLR-10	(i) TLR2/1, TLR2/10 complexes recruit the proximal adaptor MyD88	(i) Receptor complex requires TLR-2 for innate immune recognition (ii) Related to TLR1 and TLR6 (iii) Mediate immune responses to a variety of microbe and fungi	(i) Proximal adaptor MyD88 to the activated receptor complex (ii) Without a defined agonist alone or in cooperation with TLR2	(i) TLR-induced signaling, including NF-*κ*B-, IL-8, or IFN-beta-driven reporters
